# Exploring the Impact of Catechins on Bone Metabolism: A Comprehensive Review of Current Research and Future Directions

**DOI:** 10.3390/metabo14100560

**Published:** 2024-10-18

**Authors:** Iris Jasmin Santos German, Sandra Maria Barbalho, Jesus Carlos Andreo, Tereza Lais Menegucci Zutin, Lucas Fornari Laurindo, Victória Dogani Rodrigues, Adriano Cressoni Araújo, Elen Landgraf Guiguer, Rosa Direito, Karina Torres Pomini, André Luis Shinohara

**Affiliations:** 1 Department of Biological Sciences (Anatomy), School of Dentistry of Bauru, University of São Paulo, (FOB-USP), Alameda Doutor Octávio Pinheiro Brisolla, 9-75, Bauru 17012-901, Brazil; 2 Postgraduate Program in Structural and Functional Interactions in Rehabilitation, University of Marilia (UNIMAR), Marília 17525-902, Brazileleng@unimar.br (E.L.G.);; 3 Research Coordination, UNIMAR Charity Hospital, Universidade de Marília (UNIMAR), Marília 17525-902, Brazil; 4 Department of Biochemistry and Pharmacology, School of Medicine, University of Marília (UNIMAR), Avenida Hygino Muzzy Filho, 1001, Marília 17525-902, Brazil; 5 Department of Biochemistry and Pharmacology, School of Medicine, Faculdade de Medicina de Marília (FAMEMA), Marília 17519-030, Brazil; lucasffffor@gmail.com (L.F.L.);; 6 Laboratory of Systems Integration Pharmacology, Clinical and Regulatory Science, Research Institute for Medicines, Universidade de Lisboa (iMed. ULisboa), Av. Prof. Gama Pinto, 1649-003 Lisbon, Portugal; rdireito@ff.ulisboa.pt

**Keywords:** catechins, bone metabolism, bone diseases, osteoblasts, bone resorption

## Abstract

**Background/Objectives:** Degenerative musculoskeletal diseases represent a global health problem due to the progressive deterioration of affected individuals. As a bioactive compound, catechins have shown osteoprotective properties by stimulating osteoblastic cells and inhibiting bone resorption. Thus, this review aimed to address the mechanism of action of catechins on bone tissue. **Methods:** The search was applied to PubMed without limitations in date, language, or article type. Fifteen articles matched the topic and objective of this review. **Results:** EGCG (epigallocatechin gallate) and epicatechin demonstrated action on the osteogenic markers RANKL, TRAP, and NF-κβ and expression of BMPs and ALP, thus improving the bone microarchitecture. Studies on animals showed the action of EGCG in increasing calcium and osteoprotegerin levels, in addition to regulating the transcription factor NF-ATc1 associated with osteoclastogenesis. However, it did not show any effect on osteocalcin and RANK. Regarding human studies, EGCG reduced the risk of fracture in a dose-dependent manner. In periodontal tissue, EGCG reduced IL-6, TNF, and RANKL in vitro and in vivo. Human studies showed a reduction in periodontal pockets, gingival index, and clinical attachment level. The action of EGCG on membranes and hydrogels showed biocompatible and osteoinductive properties on the microenvironment of bone tissue by stimulating the expression of osteogenic growth factors and increasing osteocalcin and alkaline phosphate levels, thus promoting new bone formation. **Conclusions:** EGCG stimulates cytokines related to osteogenes, increasing bone mineral density, reducing osteoclastogenesis factors, and showing great potential as a therapeutic strategy for reducing the risk of bone fractures.

## 1. Introduction

Degenerative bone disorders impact the health system and the economy [1]. For patients, it affects the quality and quantity of bone mass and increases the risk of fractures [2]. Bone is a mineralized connective tissue that is highly vascularized. It is the largest calcium and phosphate reservoir, participating in the body’s homeostasis [3]. Bone remodeling involves many signaling mechanisms and a dynamic among the cells that participate in the turnover process [4].

Several functions characterize the bone tissue, such as locomotion, protection of organs, physical support, ion reservoir, and metabolic exchange. Phosphate and calcium are the main constituents of bone minerals [5]. Among the cells responsible for maintaining bone architecture are osteoprogenitor cells, osteoblasts, osteoclasts, and osteocytes, which play specific roles in the formation, resorption, and maintenance of bones [5]. Osteoblast and osteoclast dynamics occur under normal conditions. However, such balance is destabilized in the presence of pathologies, advanced age [3], biomechanical forces [5], mechanical stimuli, and hormonal, metabolic, local, and nutritional factors, which may regulate bone turnover [3].

Specifically, osteoclasts have a role in bone resorption and the stimulation of cytokine release, which has an effect on other types of cells [5]. Bone remodeling involves the activation and fusion phase of osteoclasts mediated by the receptors RANKL and osteoprotegerin (OPG), IL-1, calcitonin, vitamin D, and parathyroid hormone. Bone resorption takes 2–4 weeks; in contrast, the bone replacement phase involves the participation of TGF-B and bone morphogenic proteins (OPG), inhibiting the osteoclastic activity via RANK. Such a replacement phase may take from 4–6 months [5]. This process involves communication between cells and the dynamics of osteoclasts secreting acidic phosphatase, cathepsin K, and metalloproteinase-9, forming the Howship gap and reabsorbing the bone tissue. This is followed by bone formation by osteoblasts and the participation of osteocytes and coating cells, resulting in the production of new osteons [5].

Osteometabolic diseases affect bone mineral density and increase osteoclastogenesis, such as osteoporosis, a bone disease associated with advanced age that compromises the quality of life of the affected patients [5]. Nutritional strategies are required to mitigate the damage to the bone mass and reduce the risk of fractures [2].

**Figure 1:**
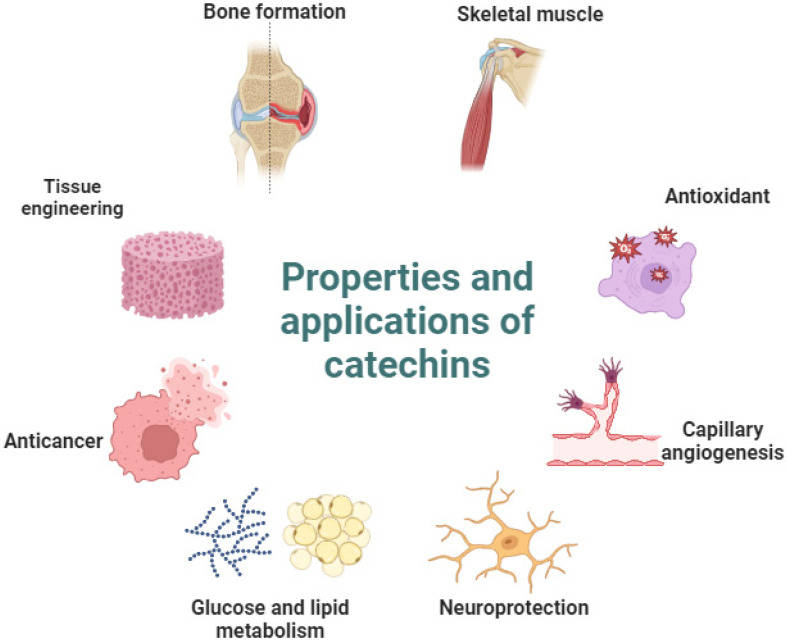
Therapeutic effects of catechins. Catechins maintain a balance in the synthesis and degradation of proteins and reduce oxidative stress by removing excessive free radicals, promoting the protection of vascular endothelial growth factors in the neovascularization process, and increasing neural function and regeneration by stimulating the binding of nerve growth factors and high-affinity receptors such as the tropomyosin kinase A (TrkA) receptor. In glucose metabolism, catechins increase the phosphorylation of PI3K and protein kinase B, in addition to controlling the oxidation of fatty acids in mitochondria. The anticancer effect occurs by inducing apoptosis of tumor cells and reducing the harmful effects of cancer treatment. Catechins stimulate bone formation mainly through suppressing the RANKL/RANK pathway. As scaffolds or as a supplement incorporated into nanoparticles, polyphenols modify the microenvironment by decreasing free radicals and reducing microorganisms and the inflammatory response.

Flavonoids are phenolic compounds with antioxidant, anti-inflammatory, and antibacterial properties in several fruits and vegetables. In this context, catechins are polyphenols obtained from *Camellia sinensis* leaves, mainly green tea [6,7]. In general, catechins show beneficial effects on skeletal muscles by suppressing genes associated with muscle atrophy and also by activating myogenic factors [7]. As antioxidant catechins can eliminate free radicals and interfere with reactive oxygen species formation [8], the role of catechin on the capillary function includes activation of VEGF and increasing the capillary number [7]. The regulation mechanism on nerve regeneration is related to an increase in the activation of the nerve growth factor (NGF) and inhibition of the degradation of motor neuron survival (SMN1), a protein associated with muscle atrophy [7]. In particular, epicatechin gallate has been shown to increase glucose absorption and accelerate fatty acid oxidation [7]. The anticancer effect of catechin is linked to the inhibition of migration and suppression of tumor activity [8]. Catechins have also been applied in tissue engineering as a delivery with different biomaterials, such as polycaprolactone and electrospun nanofibers, and exhibited significant oxygen radical decomposition [9]. Finally, bone formation can benefit from catechin by inhibiting osteoclast formation and increasing the expression of alkaline phosphatase (ALP) and osteogenic markers [8,9,10,11]. Figure 1 shows the main effects of catechins and their therapeutic applications.

Among the catechins are epicatechin (EC), epicatechin gallate (ECG), epigallocatechin (EGC), and epigallocatechin gallate (EGCG). Specifically, EGCG represents 50% of green tea and is considered a polyphenol that generates bioactive effects; it has a chemical structure of o-dihydroxycatechol in ring B and 4-oxo in ring C, favoring its antioxidant action [12]. However, all of them present biological activities that benefit health [7].

In terms of catechin metabolism and elimination, the administration of 1.5, 3.0, and 4.5 g of solid tea diluted in 500 mL of water led the volunteers to present plasma concentrations of 326 ng of EGCG, 550 ng of EGC, and 190 ng/L (nanogram/liter) of EC; the highest concentration of EGCG was found in the intestine and EC in the kidney, and elimination corresponded to 5.5–5.5 h for EGCG and 2.5–3.4 h for EGC. However, in animals, the concentrations of EGC and EC are greater than those of EGCG [11]. In addition, Tachibana (2011) [12] observed that the maximum plasma concentration was 1.5–2.5 h; after 24 h, it was not found in the plasma, and after 8 h, 90% of ECG and EC was excreted.

**Figure 2:**
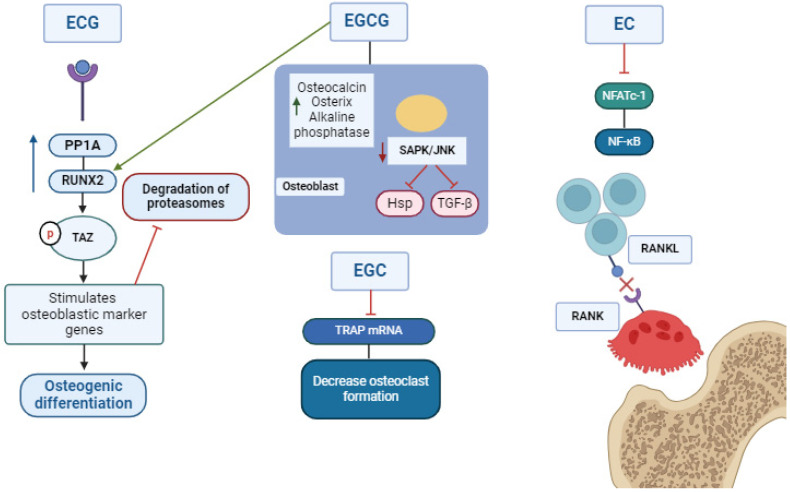
Molecular pathways of ECG, EGCG, EC, and EGC in bone tissue. The main mechanism of action of ECG stimulates the transcription of the osteogenic markers PP1A, TAZ, and Runx2, thus promoting osteogenic differentiation. EGCG increases the production of the bone markers osteocalcin, ALP, and osterix in osteoblasts and inhibits SAPK/JNK by suppressing the HSP2 and TGF-b pathways. EC also promotes osteogenesis by blocking NFATc-1, NF-κβ, and IL-1b, inhibiting the binding of RANKL and RANK, one of the main pathways of osteoclastogenesis. Finally, EGC initiates the depression of TRAP, a marker of bone resorption that promotes osteoclast migration and also degrades osteonectin. Protein phosphatase 1 catalytic subunit alpha: PP1A; runt-related transcription factor 2: RUNX2; transcriptional coactivator with PDZ-binding motif: TAZ; stress-activated protein kinase: SAPK/Jun amino-terminal kinase: JNK; heat shock protein family: Hsp; Transforming growth factor beta: TGF-β; tartrate-resistant acid phosphatase: TRAP; nuclear factor of activated T cells 1: NFATc-1; factor nuclear kappa B: NF-κβ; receptor activator of nuclear factor kappa-Β ligand: RANKL; receptor activator of nuclear factor kappa-Β: RANK.

In addition to the effects of catechins on myoblast differentiation, their importance has also been described in osteogenesis [12,13,14]. In the case of ECG, the stimulation of osteoblast differentiation occurs through increased interaction of the genetic markers PDZ (TAZ) and runt-related transcription factor 2 (RUNX2) [15]. ECG acts through PP1A (protein phosphatase 1A), an important protein in osteoblast activation and differentiation that stimulates the transcriptional coactivator [15]. On the other hand, EGCG, in addition to stimulating Runx2, osteocalcin, osterix, and alkaline phosphatase [16,17,18,19], also inhibits SAPK/JNK in osteoblasts by blocking HSP27 through TGF-β. However, it does not affect Smad2 suppression, a transcription factor that regulates myostatin. In addition, EGCG does not inhibit p38 MAP and ERK1/2 phosphorylation [17]. Concerning epicatechin, RANKL and NFATc-1 occur, reducing osteoclastogenesis induced by NF-κβ [18]. Finally, epigallocatechin increases bone mineralization by NF-κβ [18] and by inhibiting RANKL and TRAP, decreasing the formation of osteoclastic cells and increasing ALP activity [20,21,22]. Figure 2 presents the main signaling pathways of catechins.

The signaling of catechins has been investigated according to their anti-inflammatory, antioxidant, and regenerative properties, which benefit the bone turnover process by inhibiting osteoclastogenesis and stimulating osteoblastic cells. However, there is insufficient evidence in the literature from longitudinal studies, specifically in humans, to clarify the effects of catechins on BMD. Thus, this review aimed to explore the molecular and therapeutic effects of catechins in human, animal, and in vitro studies on bone tissue remodeling.

## 2. Material and Methods

The search was carried out in PubMed, websites, and citation search databases without restriction on publication date. We conducted this review using the Mendeley software (Version 2.118.0, Elsevier Ltd., Amsterdam, The Netherlands, 2024) to manage references after a thorough search for articles and analysis of titles and abstracts. Finally, 50 articles related to the topic of this review were included for analysis (14 in vitro studies, 18 in vivo articles, and 18 human studies).

## 3. RANK/RANKL/OPG Signalizing

The RANKL/RANK/OPG system regulates bone remodeling under normal and pathological conditions [20]. The oldest bone is removed during bone formation to prevent microlesions from being produced [5]. In this phase, the macrophages, derived from blood monocytes, are activated and regulated by osteoprotegerin (OPG), IL-1, calcitonin, vitamin D, and NF-κβ [5]. Osteoblastogenesis is associated with the signaling pathways Wnt, runt-related transcription factor 2 (Runx2), and osterix. At the same time, osteoclastogenesis is regulated by RANK/RANKL and RANK-L/osteoprotegerin bonding, with the participation of inflammatory cytokines that increase the osteoclastic activity, such as IL-6, IL-1, and TNFα [21,22,23].

The resorption process followed by bone replacement is known as “coupling” [24]. Osteoblasts and osteocytes are responsible for RANKL expression on the membrane surface. Moreover, osteoprotegerin (OPG) is a RANKL receptor that contributes to osteoclastogenesis regulation [23]. In addition, other genes, such as Msx and Dlx, are essential for the proliferation and differentiation of many cell lines. Thus, Msx (msh homeobox homologue-2) has a critical role in craniofacial formation [25], and Dlx-5 (distal-less homeobox-5) is an activating protein expressed in mineralized tissues that stimulates BMP-2, participating in bone formation [25]. An important marker expressed in osteoblasts and required in osterix expression and osteoblast differentiation is Runx2/Cbfa1 (transcription factor related to Runt-2/core-binding factor α_1_), which induces osteocalcin production in C2C12 and C3H10T1/2 cells and is present in the periosteum of endochondral ossification [26].

On the other hand, osteoclastogenesis, CSF-1 (for colony-stimulating factor-1), and RANKL activate a number of osteoclastic genes, such as tartrate-resistant acid phosphatase (TRAP), calcitonin receptor, and cathepsin K, allowing the maturation of osteoclasts [27].

Nutritional strategies using antioxidants have been proposed for osteoporosis patients due to their properties and action in bone metabolism and promising therapeutic potential [28]. Polyphenols, as anticatabolic agents, block oxidative stress. At the molecular level, they increase type I collagen, alkaline phosphatase, and RANKL/OPG and decrease acidic phosphatase, cathepsin K, protease, and sclerostin; at the cellular level, they increase osteoblast differentiation, decrease osteoclast viability, and reduce osteocyte apoptosis [28]. The extract is rich in catechins and increases several biomarkers, such as osteoprotegerin, bone morphogenetic protein, type 1 collagen, NF-κβ, and interleukin-6 [29].

EGCG promotes bone regeneration by modulating recruitment and macrophage activity, reducing the inflammatory process, promoting revascularization [30,31,32,33], and promoting osteogenic activity [31].

As antioxidants, catechins prevent reactive oxygen species (ROS) and, consequently, oxidative stress, which causes apoptosis of osteoblasts and osteocytes, affecting mineralization and favoring osteoclastogenesis [28,34,35].

## 4. Catechin-Based Concentration: In Vitro Studies

Some in vitro studies have evaluated the action of catechins on the primary bone markers and osteogenic signaling pathways (Table 1). The results have identified different dosages and effects of catechins on cell viability, proliferation, and their action on bone cells. In the study by Takai [32], EGCG 30 µM did not affect the cell number. However, dosages above 30 µM impaired the proliferation of MC3T3-E1 cells and showed a decrease of IL-6 60 min following the application of EGCG (30 µM). Also, Kuroyanagi [33] demonstrated a significant increase in osteoprotegerin after 48 h in MC3T3-E1 cells.

**Table 1:** Catechin activity on bone (culture cells).

Experimental Design	Type of Polyphenol	Main Results	Author/Year
Osteoblasts were obtained from calvariae of newborn ddY mice at 6–9 weeks of age.	25–100 µM EGCG for 24 h.	Decreased osteoclastic cells.	[34] Nakagawa et al., 2002 PMID: 11890677.
Osteoblast cells from the calvaria. ICR (Samtako Inc., O-San, Kyung-gi-Do, Republic of Korea).	EGCG 20, 50, and 100 µm (48 h). (Calbiochem, La Jolla, CA, USA).	Did not show action on the viability of osteoblastic cells at the concentration of 20 µm. Decreased the osteoblastic cells by 32%.	[15] Yun et al., 2004 PMID: 15324350
D1 cell culture.	EGCG 1 and 10 μmol/L (48 h).	EGCG increased the activity of ALP and osteogenic genes associated with bone mineralization.	[16] Chen et al., 2005 PMID: 16170444.
Human osteosarcoma SAOS-2 cells.	Green tea polyphenol (GTP) 24 h. Mitsui Norin Co. (Polyphenon-E^®^, Tokyo, Japan).	GTP induced apoptosis of malignant cell lines.	[36] Hafeez et al., 2006 PMID: 16797629.
Clones similar to osteoblasts MC3T3-E1 cells.	EGCG 10 and 100 µM for 48 h. Calbiochem-Novabiochem Co. (La Jolla, CA, USA).	Activated SAPK/JNK in osteoblasts and VEGF.	[37] Tokuda et al., 2007 PMID: 17031857.
MC3T3-E1 cells similar to osteoblasts.	100 µM EGCG 48 h. Calbiochem-Novabio-chem Co. (La Jolla, CA, USA).	Decreased p44/p42 MAP. In MC3T3-E1 cells, EGCG presented a reduced effect on IL-6 synthesis; however, in the primary culture of mouse osteoblasts, 30 µM of EGCG significantly reduced IL-6.	[38] Tokuda et al., 2007 PMID: 17350626.
MC3T3-E1 cells similar to osteoblasts.	EGCG (1 and 30 μM) for 24 h. Calbiochem-Novabiochem Corp. (La Jolla, CA, USA).	EGCG presented a partial effect on PDGF and did not show any action on osteocalcin and osteoprotegerin.	[32] Takai et al., 2008 PMID: 19148296.
Bone marrow cells of rats at 5 months of age.	EGCG 1–50 µM for 48 h. Sigma-Aldrich (St. Louis, MO, USA).	EGCG inactivated RANKL through the JNK/c-Jun and NF-κβ signaling pathways.	[39] Lee et al., 2010 PMID: 19828731.

Another study evaluated EGCG at a concentration of 10 µM and found it did not decrease the number of osteoclasts in the primary culture. However, 100 µM of EGCG for seven days inhibited the cell–cell fusion of pre-osteoclastic cells [40]. The study of Nakagawa [34] showed a dose-dependent decrease in osteoclasts at concentrations of 12.5–100 µM. At a concentration of 100 µM, the reduction started after 12 h of treatment with EGCG without affecting osteoblastic cells.

Also, Lin [41] investigated the effects of EGCG on RANKL activation and NF-κβ in murine pre-osteoclastic cells at concentrations of 10 and 20 µM with a progressive reduction of 17% and 32%, respectively. Furthermore, at concentrations of 50 and 100 µM, the decrease was more significant at 78% and 91%, respectively.

The EGCG (5 µM) exposure in the human osteosarcoma cell line (Saos-2) significantly increased the ALP activity within two days, and on the eighth day, it increased 35% [41]. In mesenchymal cells derived from the bone marrow of humans, EGCG treatment at 5, 10, or 20 µM improved the cell viability and BMPs, while RUNX2 were significantly regulated by EGCG at 10 µM. Additionally, Lin [42] analyzed human cells obtained from the iliac crest bone marrow of six patients between 19 and 40 years of age who underwent orthopedic surgery. EGCG (1 and 10 µmol/L) increased osteocalcin expression to 86%. ALP presented the most significant EGCG increase within 14 days of 20% (1 µmol/L) and 37% (10 µmol/L), and with respect to mineralization, EGCG (1 and 10 µmol/L) increased mineralization by 43% and 76%, respectively.

## 5. Catechin-Based Diet: Studies in Animals

A few animal studies have shown the effects of catechins on bone tissue (Table 2).

Increased bone mineral density caused by green tea supplementation has been reported in studies of obese rats [43]. The benefits of polyphenol supplementation on bone microarchitecture have been reported in obese female Sprague–Dawley rats (*n* = 40) aged three months who were supplemented with green tea polyphenol (GTP) containing 46.4% EGCG, 11.2% ECG, 10% EC, 7.8% EGC, 9.6% GCG (gallocatechin gallate), and 4.4% catechin. GTP significantly increased bone parameters, suppressed serum IGF-1, and increased femoral bone mass and tibial cortical thickness [44].

Furthermore, intraperitoneal injection of 3.4 mg/kg/day of EGCG for three months prevented a decrease in bone mineral density, increased the bone volume from 18% to 27%, and increased the trabecular thickness from 0.17 to 0.22 mm, improving the bone microarchitecture [45], in addition to improving the mechanical properties of the bone tissue, accelerating bone formation, and stimulating BMP-2 expression, thus providing bone fracture regeneration [46]. Also, the intraperitoneal injection of EGCG decreases body weight and body fat, promoting cyclin D1 expression, β-catenin, and the peroxisome proliferator-activated receptor in rats with secondary osteoporosis [47].

Shen [48] evaluated 20 F344 rats at 15 months of age, divided into untreated control (SH-C) or SH + GTP, which received GTP through the oral route. On the other hand, a group of 20 rats was only subjected to anesthesia and was randomly distributed into the control group without GTP or ORX + GTP. SH-C and ORX-C presented the lowest TRAP values. GTP suppressed TRAP only in the ORX (ORX-C vs. ORX-GTP) group. Additionally, the ORX-C group presented a lower bone mineral density. SH-C and ORX-C decreased femur quality.

Using a chronic inflammation induction model, the supplementation of 0.5% GTP in female rats at three months of age showed a significant decrease in TRAP. However, there was no action on osteocalcin [49]. This was similar to the study conducted by Shen [50], which showed that GTP significantly increased the bone mineral density of the femur and decreased TRAP. However, it was not able to change the osteocalcin levels.

EGCG was also analyzed in gelatin-based biomaterials and was able to attenuate MMP-2 by 70% and MMP-9 by 69%, in addition to increasing bone formation in 9 mm critical defects in rat calvaria, increasing osteogenesis at the implantation site after four weeks [51].

**Table 2:** Catechin activity on bone (animal studies).

Experimental Design	Type of Polyphenol	Main Results	Author/Year
Male DBA/1 mice (6–7 weeks old).	EGCG (20 µg/gm por peso corporal) (Sigma (catalog no. E4143; St. Louis, MO, USA).	EGCG decreased TRAP and osteoclast gene expression. EGCG decreased NF-ATc1; however, it could not reduce NF-κβ and c-Jun.	[52] Morinobu et al., 2008 PMID: 18576345.
Albino female rats at the age of 95–100 days.	Black tea extract (BTE), processed by Tocklai Experimental Station, Jorhat, Assam, India.	BTE increased bone density and calcium and potassium levels in ovariectomy-induced female rats associated with bone loss.	[53] Das et al., 2004 PMID: 15228990.
Twenty-one female mice (20–24 weeks old).	Black tea extract (BTE) for 28 days processed and supplied by Tocklai Experimental Station, Jorhat, Assam, India.	BTE increased the estradiol levels, restoring bone density and the calcium and phosphorus levels.	[54] Das et al., 2005 PMID: 15996685.
Eighteen albino Wistar female rats at the age of 6 months.	Black tea extract (BTE) processed by Tocklai Experimental Station, Jorhat, Assam, India.	BTE reduced the osteoclastic factors and the cytokine levels associated with bone resorption.	[55] Das et al., 2009 PMID: 19277962.
Wistar rats at the age of 4–5 months, both genders.	Black tea extract (BTE) supplied by Tocklai Experimental Station, Jorhat, Assam, India.	The BTE group presented higher levels of OPG and a reduction in RANKL activity.	[56] Karmakar et al., 2011 PMID: 21772972
Female rats, 14 months of age.	Green tea polyphenol (GTP). The dried leaves of *Camellia sinensis* were prepared daily.	GTP increased the bone mineral density of the femur.	[57] Shen et al., 2008 PMID: 18084689.
Twenty-four C56BL/six obese males, 4 weeks of age.	Green tea extract (GTE) 1 and 2% for 6 weeks. GTE: 5.6 mg caffeine/100 mg, 48% epigallocatechin gallate (EGCG), 31% epigallocatechin, 13% epicatechin gallate, and 8% epicatechin.	GTE reduced the body mass but reduced the cortical and medullary bone in thin and obese rats.	[58] Iwaniec et al., 2009PMID: 19710162.
Seventy female F344 × BFN1/NIA rats at 14 months of age.	Green tea polyphenol (GTP) for 16 weeks. GTP, 0.1% GTP, and 0.5% GTP.	Both concentrations of GTP increased the bone mineral density.	[59] Shen et al., 2009 PMID: 19118658.
Female C57BL/6J rats, six weeks of age.	Catechin 30 mg/kg (Sigma-Aldrich, MO, USA)	Catechin suppressed the osteoclast expression but with no effects on RANK.	[60] Sugawara et al., 2024 PMID: 38295903.

## 6. Catechin-Based Diet: Studies in Humans

The bone microarchitecture deterioration caused by osteoporosis is a public health concern [61]. Bone mineral density measures are a tool to prevent fracture risk [62].

Clinical studies provide important data to determine whether catechin supplementation correlates with increased bone mineral density (Table 3). The prospective study by Chen [63] investigated the relationship between regular tea consumption and bone mineral density and fracture risk. Women aged between 50 and 79 in the postmenopausal period were studied. Participants were classified according to tea consumption: less than 1 cup/day, 2–3 cups/day, 4–5 cups/day, and 6 or more cups/day. The bone mineral density of the spine and hip was analyzed. Consumption of 2–3 cups per day has been linked to greater spinal bone mineral density.

In a prospective study, Wu [64] evaluated 497 men and 540 women aged 30 in Taiwan. Regular consumers were considered as those who drink tea at least once a week for six months. The results showed that 48% were regular consumers with an average duration of 10 years of consumption. Participants who consumed tea between 6 and 10 years had a higher lumbar bone mineral density. However, consumers who had consumed tea for over 10 years had the best bone density in all measures analyzed.

Another study from China analyzed 703 participants (67% women, with an average age of 93 years and a history of osteoporosis). Women who consumed alcohol had a higher prevalence of fractures, and exercise decreased the prevalence of fractures associated with osteoporosis. However, male participants did not show significant differences [65]. In a cross-sectional study, 830 Iranian men and women aged 20 to 76 were classified as habitual tea consumers (more than 5 cups per day) and non-consumers (less than 5 cups per day). Women who consumed tea had higher bone density in the hips and a lower frequency of osteopenia and osteoporosis [66].

Kyriazopoulos [67] also did not positively correlate tea consumption with the increase in bone mineral content (BMC) and bone mineral density (BMD) of 300 healthy men (18–30 years old).

Some studies have associated the effect of tea consumption on postmenopausal women’s BMD. A cross-sectional study on 62 women consuming 30 mL of tea per day was positively associated with lumbar spine BMD [68]. In another cross-sectional study on 632 women aged ≥60, consumers of more than five cups per week had higher lumbar spine BMD [69]. Similar to these results, the cross-sectional study by Hoover [70] evaluated 62 postmenopausal women in Canada, and there was a positive correlation between tea consumption and BMD in the lumbar spine and femoral head. Contrary to this study, Vestergaard [71] associated tea intake with improvements in BMD of the femoral head, but tea consumers did not achieve significant changes in BMD of the lumbar spine.

A multicenter study on 724 postmenopausal women and tea drinkers examined the osteoprotegerin levels through BMD by densitometry, and tea consumption did not show a statistically significant increase in osteoprotegerina [72]. However, a prospective study on the Scottish population of 3000 women aged 45–54 analyzed BMD of the femoral head and lumbar spine, and the results negatively associated the flavonoid diet with markers of bone resorption; in addition, there was a positive relationship with BMC of the femoral neck and lumbar spine [73].

Jha [74] reported that tea consumption (≤1 cup/day or >1 cup/day) in 100 patients (43 men and 57 women) was associated with a significant risk of fracture. However, a case study in Canada evaluated the relationship between tea consumption (<3 cups per day or ≥3 cups per day) and the risk of hip and wrist fractures in postmenopausal women aged 50–84, and tea consumption was unrelated to fracture risk [75]. Similar to these results, Zeng [76] found that tea consumption did not positively correlate with the risk of hip fracture. Also, Tavani [77] analyzed 279 cases of hip fracture in women aged 19–74 years. Their findings showed no relationship between bone fracture and tea consumption.

**Table 3:** Catechin activity on bone (human studies).

Experimental Design	Type of Polyphenol	Main Results	Author/Year
Women and men of 50 years old.	Tea	There was a 30% decrease in the risk of hip fracture in both genders. *p* < 0.05	[78] Johnell et al., 1995 PMID: 8592959.
Women aged 65–76 years old divided into two groups: non-consumers of tea and tea consumers (1–3 glasses, 4–6 or more than 6 glasses).	Tea	The bone mineral density measurements of the greater trochanter and Ward triangle were higher in the tea consumer group; however, the femoral neck did not present a significant difference between the groups <0.05.	[79] Hegarty et al., 2000 PMID: 10731510.
Patients age 20–76 years, *n* = 1200, divided into a regular consumption group (five or more glasses of tea a day) and a control group (less than five glasses of tea a day). 10 mL of blood was collected. The BMD was analyzed.	Tea	The spine and hip BMD were higher in women (4.54% and 4.2%, respectively). In men, there were no statistical differences. Vitamin D, parathyroid hormone, and calcium presented similar values in the groups and genders.	[66] Hossein-Nezhad et al. 2007 PMID not found.
One hundred and seventy-one women in the postmenopausal period. Groups: - GTP 500 mg - Placebo + TC: (60 min, 3× a week) - GTP (500 mg daily) + TC (60 min of exercises 3× a week).	GTP capsules. Supplied by Zhejiang Yixin Pharmaceutical Co., Ltd., Jinhua, China (GTP IND no. 77,470 by FDA of USA).	The EGCG and ECG concentrations were higher in the GTP and GT + TC groups after 1 month of research and remained high 6 months after the intervention. GTP + TC did not present a time–dosage relationship. *p* = 0.05	[80] Qian et al., 2012 PMID: 23118932
Thirty patients with mild to moderate chronic periodontitis were randomly distributed into 2 groups: test (toothpaste with green tea + hygiene instruction) and control (toothpaste with fluorine and triclosan).	Green tea extract 60–90% of EGCG (Infra drug industries, Bangalore, India).	After 4 weeks of treatment, the gingival index presented significant differences: in the test group, it was 0.88, and in the control group, it was 0.54. The bleeding significantly decreased from 84.38% to 25.5% in the test group and from 78.12% to 31.25% in the control group. The test group’s depth was higher than 4 mm, with a depth of 5.38 mm and a decrease of 3.89 mm. In the control group, it was 5.34 mm, which decreased to 0.51 mm.	[81] Hrishi et al., 2016 PMID: 25690541.

## 7. Catechin in Periodontal Tissue

Periodontal disease is an inflammatory manifestation of multifactorial origin that affects the tooth’s supportive and protective tissues [82]. Its progress and clinical characteristics are determined by the individual’s immune response and interaction with the biofilm [83].

Polyphenols have been considered an adjuvant therapy to the conventional periodontal treatment protocol [84]. Several mechanisms regulate bone resorption in periodontitis, such as macrophages derived from monocytes, TNF-α, PGE_2_, and IL-1, with the activation of T, B, CD4+, and CD8+ cells, which consequently destroy the gingival microflora and accelerate bone loss; periodontal ligament cells and gingival fibroblasts participate in this [85].

The therapies for periodontal disease include surgical techniques, non-surgical procedures, and pharmacotherapy [86]. Catechins have shown inhibitory properties of pro-inflammatory cytokines, interleukins IL-1β and IL-6, and tumor necrosis factor (TNF) in patients with active periodontal disease, with benefits including inflammation regression and bone resorption [87]. However, Nakamura [88] did not note significant differences in IL-1b levels and RANKL expression among groups when catechin green tea was directly injected into the gum. In a culture of *P. gingivalis* ATCC 33277 strain (20 µM), EGCG inhibited osteoclast formation and decreased the MMP-9 levels but did not decrease MMP-2 and MMP-13 [15]. Furthermore, Sakanaka [89] evaluated the anti-adherence potential of *P. gingivalis* when applied to epigallocatechin-3, and the results showed a decrease of 80%. Similar antimicrobial activity of catechins was observed by Javadkhani [90].

Cho [87] reported that the administration of EGCG (200 mg/kg) through gavage for four weeks decreased the TNF-α and IL-6 levels in rats with induced periodontitis. In addition, Hong [91] evaluated the healing process of post-extraction alveoli in dogs with bovine collagen grafts soaked with EGCG and did not note statistical differences in the control group when assessing bone neoformation parameters. However, there was a decrease in infectious cells, fibrosis reduction, and the vestibular bone table of the alveoli. EGCG 0.02% solution for 8–15 weeks decreased the IL-1β, Il-6, Il-9, IL, and 12p70 levels, while Il-17 and TNF-α were partially reduced in the gum tissue of BALB/c female mice [92]. In addition, Yoshinaga [93] studied the effects of green tea on periodontitis induced in rats. The analyzed periodontal parameters, such as inflammatory cells at the alveolar bone level, insertion loss, and RANKL activation, were significantly reduced.

Chava [94] achieved positive results with catechin green tea in reducing periodontal pockets for four weeks in patients with chronic periodontitis. There was a reduction in the gingival index, depth of the periodontal pocket, and clinical insertion level (1.91–0.20, 2.06–0.07, and 2.1–0.21, respectively). The control group obtained 1.79–0.05, 0.97–0.02, and 0.97–0.02, respectively. These results matched those of Roodgaryan [95], where the modified papillary bleeding index, clinical loss of insertion, and the gingival index showed a significant reduction in patients at the average age of 39 years old who were divided into two groups: those who received bitter chocolate (average age, 38 years old; 12 women and eight men) and a control group (average age, 39 years old; 14 women and six men).

Periodontitis is affected by diabetes mellitus because it affects the periodontal microenvironment, which impairs several pathways that regulate pro-inflammatory cytokines and osteoclastogenic factors directly related to periodontal disease exacerbation. EGCG is considered a phytochemical agent for the treatment of bone diseases. Compared with the control, its application results in less bone loss and lower expression of RANKL-positive cells in diabetic patients with statistically significant differences in osteoprotegerin expression; however, the numbers of RUNX-2-positive cells were similar between the groups [96].

## 8. Application of Catechins on Membranes, Hydrogel, and Dental Materials

Biocompatible membranes are employed in guided bone regeneration (GBR), and their primary function is to prevent the epithelium from invading the bone defect area [97]. Polyphenols have been proposed to potentialize bone regeneration due to their anti-inflammatory, antioxidant, and regenerative properties [30].

EGCG has been shown to increase the adherence of collagen fibers, the anti-inflammatory response, and bone biomaterial in bone tissue density and uniformity, optimizing the microenvironment and the bone repair process [97].

Collagen membranes modified with EGCG decrease the inflammatory factors and promote bone formation through bone morphogenetic proteins (BMPs), diminishing the osteoclast count in addition to the expression of several growth factors associated with osteogenic differentiation [98] and anti-collagenase potential [8]. A better bond with hydrogen and hydrophobic properties, which potentialize collagenase binding and inhibition, has been attributed to EGCG [30,98]. Also, matrices with EGCG present osteoinductive properties, which increase the osteocalcin and alkaline phosphatase levels [99].

A study conducted by Chu [98] evaluated the action of membranes modified with EGCG in decreasing pro-inflammatory cytokines secreted by the osteoblast during bone regeneration using conventional collagen membranes. Of the different EGCG concentrations evaluated (0.0064%, 0.064%, and 0.64%), the concentration of 0.064% showed the best mechanical and anti-inflammatory properties and cell viability.

Hara [100] evaluated gelatin sponge modified with EGCG (vhEGCG-GS) in the regeneration of critical bone defects of 9 mm in rats. The results showed a higher quantity of osteoid tissue and collagen maturation at the highest gelatin dose, which can be related to the material shrinkage.

In Sprague–Dawley rats (male 8 weeks old), a decrease in stress-induced premature senescence (SIPS) was noted when gelatin sponge with EGCG was used, consequently showing higher activity in new bone tissue regeneration [100]. In addition, it has been reported that collagen/hydroxyapatite sponges present high compatibility with EGCG [101].

Furthermore, nanoparticle membranes covered with EGCG promoted bone regeneration in 5 mm calvaria defects in adult male Sprague–Dawley rats [102]. Similar to this study, Honda [103] evaluated EGCG in Sprague–Dawley rats’ calvaria, and the morphometric analysis showed high radiopacity by forming new bone tissue.

Due to its antibacterial and anticariogenic properties, EGCG biocompatibility has been applied as an anticaries agent in dental patches, including in dental materials such as glass ionomers [104]. In titanium surfaces, ECGC (1 mg mL^−1^) + magnesium (Mg)^2+^ caused more efficient coating compared to EGCG + sodium (Na) in defects conducted in rabbit tibia, increasing osseointegration [105]. On the other hand, Shin [106] tested BMP-2−/EGCG + biphasic calcium phosphate (BCP) in the healing process and bone formation around dehiscence implants in dogs. The results showed increased bone remodeling activity in the EGCG + BMP group with higher bone–implant contact and bone density; however, there were no significant differences.

## 9. Discussion

Bone turnover is regulated by the dynamic activity of osteoblasts, osteocytes, and osteoclasts [21]. Osteogenesis is a process that involves many biological mechanisms present in health and disease conditions [107]. This study aimed to evaluate the literature concerning the osteoprotective effects of catechins, including the molecular and therapeutic effects in humans, animals, and in vitro. The results show that catechins act in osteogenic stimulation, increasing osteoblastic differentiation markers and suppressing several transcription factors that regulate osteoblastogenesis.

Among the catechins, EGCG, which is the main polyphenol in green tea [108], has been the most studied compared to EC and ECG [13,18]. EGCG promotes a more favorable environment for osteoblast differentiation, presenting more properties that benefit bone tissue [109]. EGCG was able to suppress the phosphorylation of p38, NF-κβ, and JNK [110], in addition to increasing the expression of BMPs, osteonectin, ALP osteocalcin, and runt-related transcription factor 2 [111]. However, Takai [32] did not observe any effect of EGCG on osteocalcin and osteoprotegerin levels in osteoblast-like MC3T3-E1 cells. These results were also reported by Shen [49,50], where the studies showed a more direct action in inhibiting bone resorption, confirmed by the significant decrease in TRAP and no action on bone formation.

Lin [46] reported that EGCG significantly increased bone volume and showed more significant bone matrix formation and trabecular thickness but without significant differences in mechanical properties.

In vitro studies have shown that dosages above 30 µM impair cell proliferation [33]. In contrast, concentrations of 100 µM managed to reduce osteoclasts without producing osteoblast apoptosis [34]. This significant reduction, with a dosage between 50 and 100 µM, was confirmed by Lin [112]. However, 5–20 µM EGCG also demonstrated positive effects on cell viability, expression of BMPs, and increased mineralization [41]. EGCG increased ALP [16] and significantly reduced IL-6 [38] but did not affect osteoprotegerin and osteocalcin [32]. A decrease in osteoclastic cells was also observed due to the action of catechins [34,40]. This anti-resorptive action may occur via the MSC/stromal pathway in pre-osteoblasts, causing apoptosis of multinucleated cells by a Fenton reaction [21,34].

Catechins, as antioxidant agents, can react with reactive oxygen species. As they have phenolic groups, they can donate electrons, blocking the formation of free radicals. The aromatic groups have the function of maintaining stability. This antioxidant potential will depend on the amount of hydroxyl groups, which would place the main catechins in the following order according to the phenolic components and greater antioxidant action: EGCG > ECG > EGC > EC > C [113].

The expression of Cbfa1/Runx2, OC, osterix, and ALP is essential in the regulation of osteoblast differentiation [114]. EGCG increases the expression of these bone markers, thus increasing osteogenesis, especially when using dosages in bone culture (1 and 10 μmol/L) [16]. ALP, RUNX2, OSX, and OCN inhibit the differentiation of osteoblasts, thus inactivating RANKL [115]. On the other hand, osteoprotegerin is a protein secreted by osteoblasts and acts as an antagonist of osteoclastogenesis that binds to the receptor activator of nuclear factor-B (RANK), suppressing bone resorption [116]. However, in their review, Takai [32] did not observe a significant increase.

Kuroyanagi [33] found that EGCG stimulated osteoprotegerin synthesis in osteoblasts.

Tokuda [38] and Takai [32] observed an increase in vascular endothelial growth factor (VEGF). Runx2 regulates the expression of VEGF, osteocalcin (OCN), and receptor activator of nuclear factor kappa-B ligand (RANKL) [117]. VEGF is necessary during bone repair for the regulation of angiogenesis [118]. Catechins stimulate VEGF synthesis via the p44/p42 mitogen-activated protein (MAP) kinase pathway. As observed by Tokuda [38], EGCG managed to decrease p44/p42 MAP in MC3T3-E1 cells similar to osteoblasts.

Animal studies have shown an increase in bone density [53,54,119] and elevated osteoprotegerin [56]. The main routes of application in animals are oral and gavage. One of the biggest challenges has been the inactivity of EGCG on osteocalcin [49], in addition to a reduction in NF-κβ, c-Jun, and c -Fos. ECGG, however, regulates the expression of nuclear factor of activated T cells c1 (NF-ATc1), which may suggest that a mechanism of action of EGCG would be through this pathway [52]. NF-ATc1 is a critical transcription factor in osteoclastogenesis, heart valve formation, skeletal muscle fibers, and T-cell differentiation [65].

Human studies have shown positive effects in postmenopausal women [80,119,120,121], and the benefits of tea on bone density were related to consumption for 6–10 years [64]. Also, consumption of 2–3 cups per day showed a greater increase in bone mineral density [63]. On the other hand, Hsiao [68] concluded that 30 mL of tea per day offers benefits in bone mineral density in the lumbar spine, as also observed by Muraki [69], Hoover [61], and Hardcastle [73]. However, in males, there were no significant differences in the relationship between polyphenol consumption and the risk of osteoporosis-related fractures [65]. The results of Hossein-Nezhad [66] and Kyriazopouloset [67] were also observed by Du [65]. Male tea-drinking patients aged 45–65 had no correlation with femur and lumbar spine bone mineral density. This may be associated with the fact that tea has a more specific response related to sex via an estrogen pathway [122]. One of the factors that may be related to the decrease in ROS is estrogen; the loss of this hormone can advance the process of bone weakening and aging, as it has an antioxidant property independent of the binding to estrogen receptor alpha (ERα) DNA, decreasing oxidative stress [122].

With advances in tissue bioengineering, more recently, bone scaffolds have been linked to EGCG and EGC and tested on calvaria defects in animals, showing osteoinductive properties [97,123]. These studies show a chemical capacity and a significant stimulus for bone formation, with EGCG at a concentration of 0.064% shown to increase cell viability and mechanical properties of membranes. However, Hara [100] observed a deformity of the biomaterial, which could be associated with a limitation of EGCG as a scaffold. When combined with BMP, there was greater osseointegration around the implants [94].

The anti-inflammatory action of EGCG promoted a decrease in osteoblastic activity in vivo and in vitro by reducing the expression of cytokines and pro-inflammatory factors, such as IL-6, NF-κβ, TNF-α [102]. Additionally, EGCG exhibit an interaction with collagenase reducing the activity [123,124]. Takai [32] observed that 30 µM of EGCG for 60 min reduced IL-6 expression. EGCG did not affect cell number at 30 µM, which suggests that epicatechin does not limit cell proliferation [125]. It has been reported that prolonged periods of EGCG can cause activation of osteoblastic cells and increased levels of alkaline phosphatase [126]. ECGC had an inductive action and caused osteoclastic cell death. However, EGCG at a 25–100 µM dosage did not affect osteoblastic cells in cell culture in rats aged 6–9 months [34].

Regarding the consumption of polyphenols, no significant differences were observed when tea was consumed for less than five years [64]. This may be related to the long-term effects of tea. The variety of concentrations in animal and human studies is an important issue to analyze, specifically the low absorption of EGCG in animals [14].

The studies that evaluated epicatechin showed its action in suppressing osteoclastogenesis via RANKL and epicatechin 3-O-β-D-allopyranoside NF-κβ via the RAW 264 pathway and also increased osteocalcin levels [18]. Also, epicatechin 3-O-β-D-allopyranoside (ECAP) was able to inactivate NF-κβ by preventing the phosphorylation of IκBα, in addition to inhibiting NFATc-1 [18]. NF-κβ may connect with NFATc1 (nuclear factor of activated T cells 1) and induce osteoclastogenesis [18]. These results were also observed by Lee [39]. Among the actions of catechins on bone tissue, EGCG had a more significant antimicrobial effect than epicatechin [127]. Epicatechin showed an anticlastogenic action due to the changes it produces in NF-κβ and NFAcT-1. The number of remaining OCLs was lower by 10% compared to the EGCG control (100 M). These results suggest that EGCG can directly affect osteoclasts [34]. In female rats, epicatechin at 50 and 100 mg/kg/day prevented the loss of trabecular bone number, thickness, and volume. In addition, the trabecular area with an EC of 50 mg/kg increased by 118%, and the EC of 100 mg/kg increased by 119% [18]. However, Dostal [128] did not observe significant differences in bone mineral density when green tea extract was consumed in obese patients in the postmenopausal period, although the green extract reduced the percentage of tissue fat. In the meta-analysis carried out by Turcotte [129], obesity was associated with a high risk of fractures in the ankle but not in the hip and wrist. Those results demonstrate the heterogeneity between studies, individual factors, and fracture sites.

Several signaling systems participate in osteocyte apoptosis, such as mitogen-activated protein kinases (MAPKs), c-Jun-N terminal kinase (JNK), and p38, which affect bone remodeling [28]. EGCG decreased p44/p42 MAP, protecting bone resorption by reducing IL-6 [38]. However, Hayashi [17] did not observe a decrease in p38 MAP. MAPKs participate in several biological processes, including cell proliferation, oncogenesis, the cell cycle, oxidative stress development, and cell differentiation [130]. There is a cooperation of many transcription factors that enhance OSX to produce bone matrix [26], including BMPs, which induce the maturation of osteoblasts by increasing the expression of Runx2 [131].

The results of Takai [32] showed a reduction in IL-6 after 60 min of EGCG (30 µM) application, which may be one of the ways to suppress ECGC. EGCG also did not affect PDGF-BB. This may indicate that EGCG acts by decreasing IL-6. This polyphenol also had a limited effect on SAPK/JNK and induced AKT phosphorylation [32].

Regarding RANK/RANKL/OPG signaling, WNT signaling stimulates osteogenesis by inducing BMP-2 to regulate osteoprotegerin and indirectly modulates bone resorption through the OPG/RANKL signaling pathway [132].

The bone apposition and resorption imbalance are related to several autoimmune diseases and degenerative diseases [18]. Suppression of bone resorption by EGCG may occur through the inhibition of COX-2 and prostaglandin E2, in addition to RANKL [111]. The participation of specific cytokines during osteoclastogenesis is vital for osteoclasts, such as M-CSF [18]. It has been reported that the effects of EGCG on cell apoptosis are dose- and time-dependent [133].

Additionally, polyphenols combined or not with other polyphenols showed chemotherapeutic properties in malignant cells, and the consumption of green tea (20–60 µg/mL) may be considered as a strategy for tumors such as osteosarcoma [36] because it has inhibitory effects on cancer progression by interrupting the mitochondrial pathway [133]. However, Yun [15] did not observe any inhibitory effect with a 20 µM dosage in co-culture. Therefore, the reduction in osteoclasts by EGCG may be related to another mechanism that produces osteoclast apoptosis. Moreover, Oka [40] showed that despite the suppression of MMP-9 expression in osteoclasts, theaflavin di-gallate (TFDG), an antioxidant found in black tea, could be more effective in inhibiting osteoclast differentiation. Regarding GTP, it did not affect IkB-α; however, it suppressed phosphorylation, and IKK-α and IKK-β were inhibited at a dosage of 60 μg/mL [96].

Polyphenols have anti-inflammatory action and great antioxidant potential and can reduce the number of periodontal pathogens [134,135]. Basu [134] reported that oral use of EGCG reduced several pro-inflammatory cytokines associated with periodontitis. In periodontal disease, EGCG induces osteoclast apoptosis and acts on the main periodontal pathogens, such as Porphyromonas gingivalis, one of the main periodontopathogens that invade the host tissue and can induce MPs [15]. However, Yoshinaga [93] did not observe significant differences in inflammatory cell infiltration in rats that received a topical application of green tea extract. The number of inflammatory cells in the junctional epithelium and the loss of attachment showed significantly lower results compared to the control group. However, more studies are needed to evaluate the action of EGCG on periodontitis in humans. Furthermore, OPG is negatively coordinated in periodontitis, while RANKL is upregulated [85]. These results are contrary to the studies of Nakamura [88] and Hong [91], which did not observe a decrease in IL-1b and RANKL in participants who received catechins applied to the gums. However, Cho [87] observed decreased IL-6 and TNF levels. In cell culture, EGCG managed to reduce MMP-9, but MMP-2 and MMP-13 were not reduced. In cell culture, it was observed that only EGCG (30 µM) had a partial effect on IL-6 by stimulating the fibroblast growth factor FGF-2, resulting in a 40% reduction, which may be related to the p38 pathway and p44/p42 MAP in osteoblasts [125]. Also, the main periodontal clinical parameters to evaluate gingival inflammation (probing depth and bleeding index) decreased significantly with green tea added to the toothpaste [114]. Additionally, the action of catechins on periodontal pathogens occurs through bacterial inactivation, membrane disruption, and intracellular lipid oxidation, facilitated by the fact that Gram-negative bacteria have a thin wall. Therefore, they are more susceptible to the antimicrobial mechanism of EGCG [136].

Regarding biomaterials covered with catechins, the combination of hydrogel with EGCG showed a protective action on articular cartilage and anti-inflammatory action without presenting cytotoxicity to chondrocytes when associated with the hydrogel. However, 20 μM of EGCG had cytotoxic action. This may be due to the fact that catechins are unstable [110]. According to Chu [8], high doses of EGCG can compromise the viability of osteoblast cells associated with cytotoxicity. Still, it is necessary to consider the reduction in toxicity when using a 3D hydrogel compared to a 2D one, which may be related to the covalent bond of EGCG in the hydrogel [110]. However, when adequate dosages are used, EGCG has shown cell viability [97].

In order to improve the absorption of EGCG and reduce cytotoxicity, the enzyme-mediated HA_t hydrogel has been proposed for osteoarthritis therapy [110]. EGCG added to glass ionomer had an antimicrobial action. However, the study of Hu [104] was conducted in vitro, which may be a limitation for extrapolating these results in humans. The presence of phenolic groups gives catechins an antioxidant action by eliminating reactive oxygen species (ROS), which can be beneficial in treating diseases involving oxidative processes [110].

The results of Hara [100] showed a deficient ability of EGCG to be considered as a biomaterial with scaffold characteristics. Regarding porosity, bone mineral density in vhEGCG-GS was directly proportional to the amount of gelatin applied, as it provided a scaffold for cell migration. The relationship between the increase in the mechanical properties of gelatin scaffolds was described by Chen [137]. Also, the authors highlighted the importance of the material’s porosity in bone regeneration. Contrary to this study, EGCG has shown biocompatibility in collagen/hydroxyapatite sponges Kook [101], nanoparticles covered with EGCG [102], dental materials [92], and osseointegration of implants [106].

It is essential to point out the different factors involved in the health of bone tissues, such as genetic factors, hormones, vitamin D, and calcium consumption, but mainly individual characteristics [2]. Comprehensively, catechins offer several benefits to bone health. However, more studies are still needed to evaluate the association of catechins with scaffolds in guided tissue regeneration (RTG), guided bone regeneration (ROG), and bone regenerative medicine.

One of the limitations of the analyzed studies is the inconsistency in the definition of tea consumers. Some studies define tea drinkers as consuming up to five cups of tea a day [74] and others define it as more than two cups (200 mL), while non-drinkers are defined as those who have never drunk tea or only drink 1–2 cups per day [72]. Furthermore, it is necessary to study the stability, bioavailability, and delivery methods that can offer greater safety and bioactivity of EGCG when applied in bone tissue bioengineering to increase bone microarchitecture and thus improve management in clinical practice.

## 10. Conclusions

Catechins show osteoprotective, antioxidant, and anti-inflammatory effects. Specifically, EGCG has been found to increase calcium levels and decrease TRAP, NF-ATc1, and IL-6 in vitro. It also decreases TNF and RANKL in periodontal human pockets and increases BMD in postmenopausal women. Despite the positive results described in the literature, more studies need to be conducted, particularly in humans, analyzing the real mechanism of action and the appropriate dosages to reach such biological effects, limiting adverse effects. Catechins can be considered a strategy with great potential for clinical applicability in diseases that affect bone tissue and as a therapeutic alternative in osteoarthritis and periodontitis, presenting osteogenic and osteoclastogenesis properties and contributing to the protection of bone tissue. However, it is necessary to elucidate the effect of catechins on RANK and osteocalcin levels and the stability of catechins depending on the type of delivery used and the physiological events it triggers.

## 11. Future Perspectives

There is a need to elucidate the mechanisms involving catechins’ effects in delivery systems with the aim of encapsulating catechins in biocompatible nanodelivery based on polymers, peptides, nanocompounds, or other nanocarriers with properties that optimize the low bioavailability of catechins. Polyphenols still present some barriers, representing a challenge in their clinical application.

Genetic technology and growth factors can improve catechins, enhancing their biological effects and therapeutic action in diseases that affect bone tissue. It is recommended that future longitudinal and randomized studies be developed that evaluate the bone mineral density index, specific bone biomarkers, and the effect of catechins on the influence of physical activity on bone tissue health and patients’ quality of life.

## Data Availability

No new data were created or analyzed in this study. Data sharing is not applicable to this article.
